# The influence of population size, noise strength and behavioral task on best-encoded stimulus for neurons with unimodal or monotonic tuning curves

**DOI:** 10.3389/fncom.2015.00018

**Published:** 2015-02-17

**Authors:** Stuart Yarrow, Peggy Seriès

**Affiliations:** Institute for Adaptive and Neural Computation, School of Informatics, University of EdinburghEdinburgh, UK

**Keywords:** population code, SSI, fisher information, Chernoff, tuning curve, monotonic, unimodal

## Abstract

Tuning curves and receptive fields are widely used to describe the selectivity of sensory neurons, but the relationship between firing rates and information is not always intuitive. Neither high firing rates nor high tuning curve gradients necessarily mean that stimuli at that part of the tuning curve are well represented by a neuron. Recent research has shown that trial-to-trial variability (noise) and population size can strongly affect which stimuli are most precisely represented by a neuron in the context of a population code (the best-encoded stimulus), and that different measures of information can give conflicting indications. Specifically, the Fisher information is greatest where the tuning curve gradient is greatest, such as on the flanks of peaked tuning curves, but the stimulus-specific information (SSI) is greatest at the tuning curve peak for small populations with high trial-to-trial variability. Previous research in this area has focussed upon unimodal (peaked) tuning curves, and in this article we extend these analyses to monotonic tuning curves. In addition, we examine how stimulus spacing in forced choice tasks affects the best-encoded stimulus. Our results show that, regardless of the tuning curve, Fisher information correctly predicts the best-encoded stimulus for large populations and where the stimuli are closely spaced in forced choice tasks. In smaller populations with high variability, or in forced choice tasks with widely-spaced choices, the best-encoded stimulus falls at the peak of unimodal tuning curves, but is more variable for monotonic tuning curves. Task, population size and variability all need to be considered when assessing which stimuli a neuron represents, but the best-encoded stimulus can be estimated on a case-by case basis using commonly available computing facilities.

## 1. Introduction

Mapping the response of a neuron to a range of stimuli by constructing a tuning curve or receptive field is one of the longest established and most widely used approaches in sensory neuroscience. Often the tuning curves of many neurons are distributed across the space of possible stimuli to form a population code, where information is transmitted through the combined activity of the neurons, and each neuron conveys information about a limited range of stimuli. Despite the simplicity and widespread use of tuning curves, they remain open to misinterpretation; in particular, there is a tendency for neurons to be associated by default with the stimuli that trigger their strongest responses. This is sometimes stated explicitly, but is also implicit in the language used to describe response properties, for example in the term “preferred stimulus” or when a neuron is described as being selective for a particular stimulus. While this is a convenient way to refer to tuning curves, it is not a reliable indication of what information a neuron contributes to a population code—what its *informational* tuning curve is, and hence which stimulus (or stimuli) it conveys the most information about: its best-encoded stimulus.

Even within the simplified framework of rate coding there are a number of measures that can be used to quantify the amount of information transmitted by a neuron about a specific stimulus, to construct informational tuning curves and identify the best-encoded stimuli. These measures have distinct, but overlapping scopes of application and do not always yield similar predictions as to the best-encoded stimuli, so selecting the right measure is an important step in any analysis. Here we give a brief overview of the properties and applications of three such measures: the Fisher information (Fisher, 1925), the stimulus-specific information (Butts, 2003), and the Chernoff distance (Chernoff, 1952). Mathematical definitions of all three measures are given in the Materials and Methods Section.

Fisher information (Fisher, 1925) is a measure of the precision with which a parameter (typically a stimulus in studies of neural coding) of a parametric probability distribution (e.g., a distribution of neuronal responses) can be estimated, based on a sample from that distribution (e.g., a set of neuronal responses). It has been used in both theoretical (see e.g., Paradiso, 1988; Seung and Sompolinsky, 1993; Abbott and Dayan, 1999; Wilke and Eurich, 2002; Berens et al., 2011) and experimental (Jenison and Reale, 2003; Harper and McAlpine, 2004; Durant et al., 2007; Gutnisky and Dragoi, 2008) population coding studies. Fisher information has also been used as an objective functional for synaptic weights in feedforward neural networks, where it leads to Hebbian-like learning (Echeveste and Gros, 2014). A significant limitation of the Fisher information is that it is determined entirely by local properties of the tuning curve; as such, its relevance is restricted to tasks involving closely spaced stimuli, for example fine discrimination, and it can only be applied to continuous stimulus parameters. The Fisher information defines a lower bound (the Cram’er-Rao bound) on the variance of an optimal unbiased estimator and, equivalently, the discrimination threshold. This makes it useful for estimating these quantities at the neural level, allowing comparison between neuronal and behavioral precision. However, when the true precision of the code does not saturate that bound the Fisher information can be misleading (Bethge et al., 2002; Xie, 2002; Yarrow et al., 2012); we refer to this as the pre-asymptotic regime. As the number of neurons in the population increases, the precision of the code approaches the Cram’er-Rao bound; we describe this as the asymptotic regime. Because it is a function of the stimulus and, for typical Poisson-like trial-to-trial variability, it is approximately proportional to the first derivative of the tuning curve, the Fisher information predicts best-encoded stimuli at points of maximum tuning curve gradient (e.g., the flanks of peaked tuning curves). Correspondingly, the Fisher information is zero where the tuning curve gradient is zero—this includes tuning curve peaks. One major advantage of the Fisher information is that closed form solutions are available for many noise models, so it tends to be easy to compute from parametric tuning curves and variability models, although constructing accurate parametric models from experimental data may be difficult in itself.

Information theory provides a powerful and general framework for studying neural coding; it takes into account all forms of statistical dependency (not just correlations), does not rely on assumptions about the form of probability distributions, and is not specific to any particular sensory task. The stimulus-specific information (SSI; Butts, 2003) is a decomposition of Shannon's scalar mutual information into stimulus-specific components and it quantifies the average reduction in uncertainty (entropy) resulting from the presentation of a given stimulus. As the SSI is not linked to any particular task in the way that Fisher information is, it gives a more general picture of the amount of information conveyed about each stimulus. Interestingly, theoretical studies of population codes based on bell-shaped unimodal tuning curves have shown that the best-encoded stimulus can coincide with either the peak of the tuning curve or, as predicted by the Fisher information, the sloping flanks. The best-encoded stimulus predicted by the SSI is determined both by the level of trial-to-trial variability (noise) and by the number of neurons in the population (Butts and Goldman, 2006; Yarrow et al., 2012). Evaluating the SSI is much more computationally intensive than computing the Fisher information, but it has recently been shown that the SSI can be computed for population codes involving hundreds of neurons using a standard desktop computer (Yarrow et al., 2012). Perhaps partly due to its computational complexity, the SSI has until now been employed in only a small number of experimental studies (e.g., Sawtell and Williams, 2008; Remedios et al., 2009; Montgomery and Wehr, 2010).

The Chernoff distance (Chernoff, 1952) is a measure of the difference between two probability distributions. In the study of neural codes, it can be used to quantify the amount of overlap between the response distributions associated with two stimuli, and hence the ease with which the two stimuli can be discriminated. The Chernoff distance is linked to the mutual information between stimulus and response and also to the error rate in a two-alternative discrimination task (Kang and Sompolinsky, 2001). Although the Chernoff distance has been used to quantify the precision of population codes as a function of the distance between stimuli in a discrimination task (Kang et al., 2004), it has not previously been used to predict best-encoded stimuli; in this article we explore the latter application of the Chernoff distance. Computing the Chernoff distance for many parametric distributions is faster than computing the SSI, making it a potentially useful method of determining best-encoded stimulus in two-alternative tasks, but it does involve iterative optimization, which accounts for much of the computational effort. However, a recently described information geometric approach (Nielsen, 2013) greatly reduces the computational complexity of the optimization, and we show how this method can be used to efficiently compute the Chernoff distance for populations of Poisson neurons.

Several factors—both neuronal and environmental—affect the best-encoded stimulus. Of the neuronal factors, the shape of the tuning curve is perhaps the most obvious. Tuning curves are a very widely used model of neuronal activity, but their implications in terms of the quantity of information that neurons transmit about particular stimuli are still not fully understood. While bell-shaped tuning curves have been widely studied (see e.g., Paradiso, 1988; Zhang and Sejnowski, 1999; Sompolinsky et al., 2001; Wilke and Eurich, 2002; Butts and Goldman, 2006; Yarrow et al., 2012), sigmoidal monotonic tuning curves have received little attention from the theoretical community (Guigon, 2003; Salinas, 2006; McDonnell and Stocks, 2008). Studies using the SSI have shown that, for rich stimulus ensembles with many possible stimuli, the best-encoded stimulus for peaked tuning curves depends on the level of trial-to-trial variability and the number of neurons in the population (Butts and Goldman, 2006; Yarrow et al., 2012). Population codes involving monotonic tuning curves have received comparatively little attention, but are also important in sensory neuroscience as they are often found where the intensity of neuronal activity reflects the intensity of the stimulus, for example relative luminance in the visual system (e.g., Sakmann and Creutzfeldt, 1969), sound intensity in the auditory system (e.g., Sachs and Abbas, 1974), and pressure of touch in the somatosensory system (Adrian and Zotterman, 1926). For monotonic tuning curves, the Fisher information predicts that the best-encoded stimulus lies on the sloping flank of the tuning curve, but the SSI has not previously been computed for such tuning curves. Building upon the earlier studies of unimodal tuning curves, we first focus on monotonic tuning curves and ask: (1) what are the best-encoded stimuli for monotonic tuning curves? and (2) are the best-encoded stimuli dependent on the level of variability and the population size?

Studying monotonic tuning curves introduces some additional complexity, as monotonicity requires a linear stimulus variable rather than a circular one. Most theoretical studies of population coding (e.g., Paradiso, 1988; Seung and Sompolinsky, 1993; Zhang and Sejnowski, 1999; Wilke and Eurich, 2002; Butts and Goldman, 2006; Berens et al., 2011; Yarrow et al., 2012) use angular stimuli, as the endless nature of the stimulus space is mathematically convenient and orientation tuning in the visual cortex is a popular subject for experimental work. Whereas a uniform stimulus distribution on a periodic variable (such as edge orientation in natural scenes) can be a reasonable approximation of reality, linear stimulus parameters such as luminance or sound intensity are far from uniformly distributed in nature. The popularity of circular stimulus spaces in theoretical work has meant that relatively little is known about how non-uniformity of the stimulus distribution might affect which stimuli are best encoded by a neuron. To address this, we compute the SSI for uniform and non-uniform stimulus distributions, and show how best-encoded stimuli for both unimodal and monotonic tuning curves are affected by local non-uniformity in the stimulus distribution.

The SSI can also be used to determine the best-encoded stimulus in the context of any arbitrary task by manipulating the stimulus distribution; for example, a two-alternative forced choice task can be modeled by a stimulus ensemble consisting of only two stimuli. When the stimulus ensemble is restricted by the task in this way, the spacing between stimuli determines the best-encoded stimulus for unimodal tuning curves (Butts and Goldman, 2006). For closely spaced stimuli, the difference in the neuronal response distributions that they elicit is dominated by the tuning curve gradient and the SSI predicts that the best-encoded stimuli are on the flanks of the tuning curve. This is the same as the prediction of the Fisher information—which is unsurprising as the Fisher information is specific to fine discrimination and is based on the derivative of the tuning curve. Conversely, if the spacing between stimuli is large, the SSI predicts that the best-encoded stimulus lies at the peak of the tuning curve. Building upon this work, we ask: how does the best-encoded stimuli for monotonic tuning curves depend on the behavioral task?

In addition to addressing the open questions described above, this article aims to give an overview of how the SSI, Fisher information and Chernoff distance can be used to analyze tuning curves and trial-to-trial variability to obtain informational tuning curves and identify the best-encoded stimuli for any arbitrary task. First, we use the SSI to determine the best-encoded stimuli for populations of neurons with monotonic tuning curves and show that, as with unimodal tuning curves, the SSI and the Fisher information predict similar best-encoded stimuli for large populations. In smaller populations, we show that the best-encoded stimulus depends on the level of trial-to-trial variability. We then go on to show how Chernoff distance can be used to quickly estimate the best-encoded stimulus for two-alternative forced choice tasks, and to examine how the best-encoded stimulus for monotonic tuning curves is affected by the behavioral task, specifically the distance between stimuli and the number of stimuli in discrimination and classification tasks. We show that, as with unimodal tuning curves, when the stimulus distribution is primarily defined by the task it is the task, together with the tuning curves and variability, that determines the best-encoded stimulus.

## 2. Materials and methods

### 2.1. Model framework

Our analyses are based upon a model population of rate-coding neurons representing an abstract one-dimensional stimulus *S*. We consider both discrete and continuous stimuli and, unless otherwise stated, the stimulus is uniformly distributed across a finite non-periodic interval.

(1) $$\text{Discrete: }P(S=s)=\frac{1}{k}$$

(2) $$\text{Continuous: }p(S=s)=\frac{1}{s_{max}-s_{min}}$$

Where *P*(*S* = *s*) and *p*(*S* = *s*) are probability mass and density, respectively at the stimulus value *s* (uppercase characters represent ensembles and lowercase characters represent concrete values). In the discrete case the ensemble consists of *k* stimulus values, and in the continuous case stimuli can take any value in the interval [*s*_*min*_, *s*_*max*_].

The stimulus is encoded in the firing rates of *N* neurons with response spike counts **r** = {*r*_1_, *r*_2_, …, *r*_*N*_} (bold symbols indicate vector quantities); the responses of each neuron are conditionally independent given *S*. The characteristic stimuli of the *N* neurons are uniformly distributed across the stimulus space. The response *R*_*i*_ of the *i*th neuron is Poisson distributed with the expected value defined by the product of a tuning function *f*_*i*_(*s*) and integration time τ:

(3) $$R_{i}\sim \text{pois}\left[\tau f_{i}(s)\right]$$

The conditional response distribution for the *i*th neuron, given *s*, is therefore:

(4) $$P(R_{i}=r_{i}|S=s)=\frac{{\left[\tau f_{i}(s)\right]}^{r_{i}}\exp\left[-\tau f_{i}(s)\right]}{r_{i}!}$$

Because the responses of each neuron are conditionally independent, the population response distribution, conditioned upon *s*, is given by:

(5) $$P(R=r|S=s)=\prod_{i=1}^{N}\frac{{\left[\tau f_{i}(s)\right]}^{r_{i}}\exp\left[-\tau f_{i}(s)\right]}{r_{i}!}$$

Two forms of tuning curve are investigated in this article: Gaussian unimodal and sigmoidal monotonic. The tuning curve of the *i*th neuron in a population with unimodal tuning curves is defined as:

(6) $$f_{U}^{i}(s)=f_{bg}+f_{mod}\exp\left[-\frac{{\left(s-\sigma_{i}\right)}^{2}}{2\omega^{2}}\right]$$

Where *f*_*bg*_ and *f*_*mod*_ are the background firing rate and firing rate modulation depth (*f*_*mod*_ = *f*_*max*_ − *f*_*bg*_), both in spikes/s, σ_*i*_ is the characteristic (peak) stimulus and ω is a width parameter. The monotonic tuning curve of the *i*th neuron is similarly defined as:

(7) $$f_{M}^{i}(s)=f_{bg}+\frac{f_{mod}}{1+\exp\left(-\frac{s-\sigma_{i}}{\omega }\right)}$$

**Figure 1:**
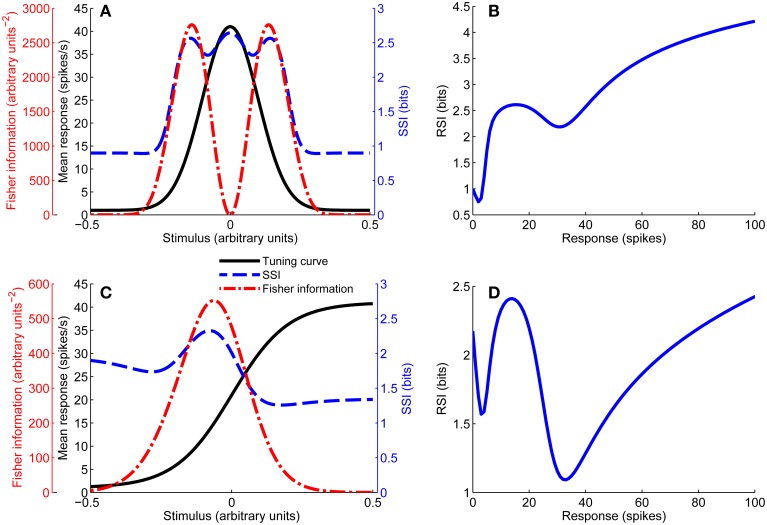
**Unimodal tuning curve for a single neuron **(A)**, together with the SSI and Fisher information**. Note that the Fisher information is greatest at the flanks of the tuning curve, while the SSI at the peak and flanks is roughly equal in this case (close to the transition between peak and flank coding regimes). **(B)** Shows the response specific information for the tuning curve shown in **(A)**. Example of a monotonic tuning curve for a single neuron **(C)**, along with its SSI and Fisher information; here both the SSI and Fisher information predict similar best-encoded stimuli. **(D)** Gives the response specific information for the tuning curve shown in **(C)**. Parameters: *N* = 1, *f*_*bg*_ = 1 spike/s, *f*_*mod*_ = 40 spikes/s, τ = 1 s, ω = 0.1, σ = 0, stimulus ensemble consists of 401 equally probable discrete stimuli regularly spaced in the interval [−1, 1].

In this case the characteristic stimulus σ is the midpoint of the sloping flank of the tuning curve. Figure 1 shows examples of both types of tuning curve.

### 2.2. Fisher information

The Fisher information *J*(*s*) for a neuron with rate tuning curve *f*(*s*), integration time τ, and Poisson variability (Seung and Sompolinsky, 1993; Bethge et al., 2002) is given by:

(8) $$J(s)=\tau \frac{f^{\prime }{\left(s\right)}^{2}}{f(s)}$$

The units of the Fisher information are A^−2^, where A are the arbitrary units of the stimulus variable. The tuning curve derivatives *f*′_*U*_(*s*) for unimodal turning curves and *f*′_*M*_(*s*) for monotonic tuning curves are given by:

(9) $${f^{\prime }}_{U}(s)=f_{mod}\frac{\sigma_{i}-s}{\omega^{2}}\exp\left[-\frac{{\left(s-\sigma_{i}\right)}^{2}}{2\omega_{i}^{2}}\right]$$

(10) $${f^{\prime }}_{M}(s)=\frac{f_{mod}}{2\omega \left[\cosh\left(\frac{\sigma_{i}-s}{\omega }\right)+1\right]}$$

Figure 1 includes examples of the Fisher information for both unimodal and monotonic tuning curves.

### 2.3. Stimulus-specific information

The response-specific information (RSI; DeWeese and Meister, 1999), also known as the specific information, is the average reduction in uncertainty about the stimulus (reduction in stimulus entropy) associated with observing a neuronal response:

(11) $$I(r)=\sum_{s\in S}p(s|r)\log p(s|r)-p(s)\log p(s)$$

(12)    =H(S)−H(S|R=r)

It is often more useful to be able to quantify the information associated with a given stimulus, rather than a response. The SSI *I*(*s*) (Butts, 2003) is the expected value of the RSI associated with a given stimulus i.e., the RSI averaged over the conditional response distribution. Examples of response-specific and SSI for unimodal and monotonic tuning curves are shown in Figure 1. The SSI is defined as:

(13) $$I(s)=\sum_{r\in R}p(r|s)I(r)$$

(14) $$\;=\sum_{r\in R}p(r|s)\left[\sum_{s^{\prime }\in S}p(s^{\prime }|r)\log p(s^{\prime }|r)-p(s^{\prime })\log p(s^{\prime })\right]$$

The precision of the modeled population codes was always set (by choosing appropriate *f*_*bg*_, *f*_*mod*_ and τ) such that the mutual information between stimulus and response did not saturate the stimulus entropy, as this would distort the shape of the SSI. We compute the SSI using the Monte Carlo method described in our earlier article (Yarrow et al., 2012). This approach removes the necessity to exhaustively integrate over the high-dimensional response ensemble and makes it feasible to compute the SSI for populations of hundreds of neurons.

### 2.4. Chernoff distance

We use the Chernoff distance (Chernoff, 1952) to quantify the dissimilarity between the response distributions elicited by two stimuli, which gives an indication of the discriminability of the stimuli. The Chernoff distance between the response distributions *P*(**r**|*s*_1_) and *P*(**r**|*s*_2_) associated with two different stimuli *s*_1_ and *s*_2_ is defined as:

(15) $$D_{C}(s_{1},\;s_{2})=\underset{\alpha \in (0,\;1)}{\max}\left[-\log\sum_{r\in R}P^{\alpha }(r|s_{1})P^{1-\alpha }(r|s_{2})\right]$$

Much of the computational complexity in evaluating the Chernoff distance arises from the need to integrate over the response ensemble for every iteration of the maximization on the exponent α. This means that, as the population size increases, it rapidly becomes very time consuming to compute the Chernoff distance. However, it is possible in many cases to avoid this expensive calculation. Firstly, a closed-form solution exists for the Chernoff distance between two univariate Poisson distributions (Johnson and Sinanović, 2001; Nielsen, 2013), here with parameters λ_1_ and λ_2_:

(16) $$\begin{array}{l} D_{C}(\lambda_{1},\;\lambda_{2})=\lambda_{1}\frac{(\Lambda -1)\left(\log\frac{\Lambda -1}{\log\Lambda }-1\right)+\log\Lambda }{\log\Lambda }\\ \text{where }\Lambda =\frac{\lambda_{2}}{\lambda_{1}}\; \end{array}$$

The multivariate case, however, is a little more complex. A recently proposed information geometric method (Nielsen, 2013) provides a way to perform an alternative optimization with reduced computational complexity; this method involves computing Bregman divergences in the natural parameter space of the distributions. Following Nielsen's univariate Poisson example (Nielsen, 2013), the natural parameters for the joint distribution of *N* independent Poisson variables with means **λ** = {λ_1_ … λ_*N*_} are **θ**(**λ**), where **θ**_*i*_(λ_*i*_) = log λ_*i*_. The Bregman divergence between two such distributions with natural parameters **θ** and **θ**′ is given by:

(17) $$B(\theta ,\;\theta^{\prime })=\sum_{i=1}^{N}\exp{\theta^{\prime }}_{i}-\sum_{i=1}^{N}\exp\theta_{i}-\sum_{i=1}^{N}\left({\theta^{\prime }}_{i}-\theta_{i})\exp(\theta_{i}\right)$$

Considering the distributions of responses to two stimuli *s*_1_ and *s*_2_, with corresponding natural parameters **θ**_1_ and **θ**_2_, there exists a point **θ** on the line joining **θ**_1_ and **θ**_2_ such that *B*(**θ**_1_, **θ**) = *B*(**θ**_2_, **θ**) = *D*_*C*_(*s*_1_, *s*_2_). The intermediate point **θ** can be expressed as a weighted average, where α controls the weighting: **θ** = α**θ**_1_ + (1 − α)**θ**_2_. Computing the Chernoff distance is thus reduced to a bisection search involving the calculation of two Bregman divergences (Equation 17) per iteration. Our implementation was verified by cross checking against directly calculated Chernoff distances for univariate test cases.

### 2.5. Quantifying similarity in the shape of information measures

It is often useful to compare the shapes of informational quantities that are both functions of the stimulus, but have different units, e.g., the SSI and the Fisher information. To do this, we first discretize the stimulus space then compute the value of both measures at each stimulus value. We can then treat the resulting discretized functions of the stimulus as vectors, where the shape of the function is equivalent to the direction of the vector. Similarity of shape can then be quantified by normalizing the vectors to unit length and taking the dot product. Using the SSI as an example, the normalized SSI $\hat{I}(s)$ is given by:

(18) $$\hat{I}(s)=\frac{I(s)}{\sqrt{\sum_{s\in S}I^{2}(s)}}$$

If, for example, the Fisher information *J*(*s*) is similarly normalized, then the normalized dot product is given by:

(19) $$\hat{I}•\hat{J}=\sum_{s\in S}\hat{I}(s)\hat{J}(s)$$

This is a scalar measure of shape similarity, where a value of one indicates that the two functions have identical shape, i.e., that they are directly proportional to one another.

### 2.6. Task modeling

Two-alternative forced choice tasks are frequently used in experimental neuroscience because of their simplicity. We modeled tasks of this type in order to find out which stimuli were best encoded by a given neuron for a given stimulus spacing Δ*s*. In our model the absolute values of the stimuli (the choices) are not fixed, only the distance between choices Δ*s* appears as a parameter. This can be considered equivalent to an experimental setup where the stimulus choices are fixed relative to one another, but their position can vary with respect to the tuning curves, such as in a visuospatial task without predefined gaze fixation or an auditory source discrimination task where the head is free to move.

Generalizing from two-alternative to *K*-alternative tasks, we assume that the stimulus ensemble consists of *K* distinct stimuli regularly spaced at intervals of Δ*s*, and that the set of *K* stimuli can be translated anywhere in the stimulus space. Any concrete stimulus value *s* can be a member of the stimulus ensemble when the ensemble is translated to *K* different positions, as *s* can be the first, second, third, etc., stimulus in the set. We denote each SSI component as *I*_*k*_(*s*), where *s* is the *k*th member of the stimulus ensemble. For example, if *K* = 2 we have two components of the SSI:

(20) $$I_{1}(s)=I(s;\;S=\{s,\;s+\Delta s\})$$

(21) $$I_{2}(s)=I(s;\;S=\{s-\Delta s,\;s\})$$

By substituting the stimulus ensemble *S* into Equation 14, we can obtain the full expression for *I*_1_(*s*):

(22) $$\begin{array}{l} I_{1}(s)=\sum_{r\in R}p(r|s)[p(s|r)\log p(s|r)-p(s)\log p(s)\\ \;+p(s+\Delta s|r)\log p(s+\Delta s|r)-p(s+\Delta s)\log p(s+\Delta s)] \end{array}$$

*I*_2_(*s*) can be similarly expanded. We assume that each of the cases is equally likely, and compute the SSI as the simple average over the *K* components:

(23) $$I(s)=\frac{1}{K}\sum_{k=1}^{K}I_{k}(s)$$

**Figure 2:**
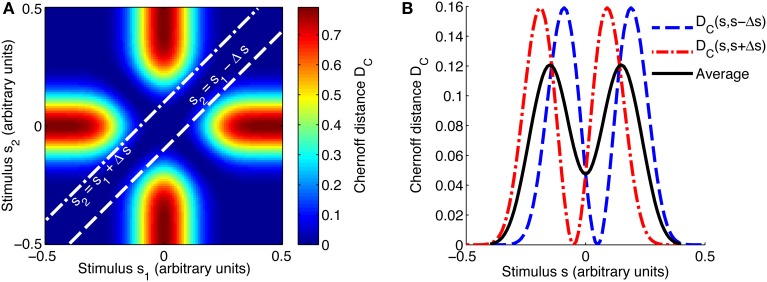
**Evaluating the best-encoded stimulus for a 2AFC task using the Chernoff distance**. **(A)** Shows a map of the Chernoff distance between the response distributions for every possible pair of stimuli. The white lines overlaid on the heat map indicate where the difference between stimuli *s*_1_ and *s*_2_ is equal to the stimulus spacing of interest, in this case Δ*s* = 0.1. In a 2AFC task with a given stimulus spacing Δ*s*, a stimulus *s* can occur in two ways: paired with *s* − Δ*s* or with *s* + Δ*s*; these two possibilities correspond to the two white lines on **(A)**. The interrupted red and blue curves in **(B)** correspond to slices through the map shown in **(A)**; *D*_*C*_(*s*, *s* + Δ*s*) corresponds to the line *s*_2_ = *s*_1_ + Δ*s* and *D*_*C*_(*s*, *s* − Δ*s*) to *s*_2_ = *s*_1_ − Δ*s*. We construct an information tuning curve (solid black line) based on the Chernoff distance by taking the average of these two components. Parameters: *N* = 1, *f*_*bg*_ = 1 spike/s, *f*_*mod*_ = 40 spikes/s, τ = 50 ms, ω = 0.1, σ = 0, stimulus ensemble consists of 101 equally probable discrete stimuli regularly spaced in the interval [−0.5, 0.5].

The same method was used to compute the Chernoff distance as a function of the stimulus (see Figure 2 for an example of how the combined Chernoff distance is computed), but here the number of stimuli in the set is fixed at *K* = 2 due to the inherent limitations of the measure.

Note that this is slightly different to the method of averaging used by Butts and Goldman. In Figure 4 of Butts and Goldman (2006), the SSI at stimulus *s* is given by $I(s)=\frac{I(s-\Delta s/2)+I(s+\Delta s/2)}{2}$, where the stimulus ensemble is *S* = {*s* − Δ*s*/2, *s* + Δ*s*/2}. As this is an average over the stimulus ensemble it is equal to the mutual information between the stimulus and responses, given that stimulus ensemble. The two methods of averaging give very similar results for small Δ*s*, but differ at larger Δ*s*. Our method of averaging over SSI components does not yield a mutual information, and is more strictly local to *s*, which makes it better suited for assessing the best-encoded stimulus when Δ*s* is large, while remaining equally valid for small values of Δ*s*.

When modeling forced choice tasks it is sometimes useful to give the interval between stimuli Δ*s* in a normalized form δ*s*, such that it is expressed relative to the width of the tuning curve flank:

(24) $$\delta s=\Delta s\frac{f_{mod}}{\max\;f^{\prime }(s)}$$

Where *f*_*mod*_ and max *f*′(*s*) are, respectively, the modulation depth and maximum gradient of the tuning curve. Δ*s* = |*s*_2_ − *s*_1_| is the stimulus interval and *s*_1_, *s*_2_ are adjacent stimuli.

### 2.7. Visualizing uncertainty in the neural code

In any probabilistic code, information is lost when symbols (here neural responses) are ambiguous, when it is unclear which stimuli caused them. When studying best-encoded stimuli and the shape of the SSI and similar measures, it is useful to be able to visualize this ambiguity; the “confusion” between stimuli that is introduced by the code. We do this by considering a hypothetical observer of the population response with no direct knowledge of the stimulus, who tries to infer *s* from the responses **r** to a single stimulus presentation. We represent the observer's knowledge of the stimulus by the random variable *Z*, and assume that the observer has full knowledge of the stimulus distribution *P*(*S*) and the stochastic encoding scheme *P*(*R*|*S*). For simplicity, we assume that the observer's prior *P*(*Z*) is equal to the true stimulus distribution *P*(*S*) (although this need not be the case; any prior could be modeled). We are interested in the distribution *P*(*Z*|*S*), as this tells us what the hypothetical observer (or, equivalently, any downstream neural information processing element) can infer on average about the stimulus, following the presentation of a given stimulus. Although there is a close relationship between SSI and the uncertainty in *P*(*Z*|*S*), it is important to note that the SSI is not directly related to the entropy *H*(*Z*|*S* = *s*). The RSI, and hence the SSI, are based upon a difference in entropies (Equation 12) and a similar stimulus-specific measure could be derived from the posterior conditional distribution: *I*_2_(*s*) = *H*(*S*) − *H*(*Z*|*S* = *s*). These measures are not the same: the SSI is based on an expected entropy, whereas the alternative measure *I*_2_(*s*) is based on the entropy of an expected distribution; the difference is in the order in which the average is taken and the entropy calculated.

*P*(*Z*|*S*) is computed as follows.

(25) $$P(z|s)=\sum_{r\in R}P(z|r)P(r|s)$$

Where *P*(*z*|*r*) = *P*(*s*|*r*), since our observer has full knowledge of the encoding process—the model is symmetrical. *P*(*s*|*r*) is obtained by applying Bayes' theorem:

(26) $$P(s|r)=\frac{P(r|s)P(s)}{P(r)}=\frac{P(r|s)P(s)}{\sum_{s\in S}P(r|s)P(s)}$$

For single neurons or very small populations the sum over *R* is straightforward, but it becomes intractable as the population size increases. To overcome this, we used a Monte Carlo method identical to that used when calculating the SSI (Yarrow et al., 2012).

### 2.8. Implementation of models and measures

All models and measures were implemented in Matlab. This Matlab code has been made publicly available as part of the Popcode toolbox (https://github.com/StuYarrow/Popcode).

## 3. Results

### 3.1. The effect of trial-to-trial variability in single neurons

#### 3.1.1. Unimodal tuning curves

In neurons with unimodal tuning curves, the best-encoded stimulus predicted by the SSI depends both on the level of trial-to-trial variability (noise) and on the number of neurons in the population (Butts and Goldman, 2006; Yarrow et al., 2012). The best-encoded stimuli according to the SSI can lie on the flanks of the tuning curves, or at the peaks of the tuning curves (in small populations with high noise or short integration times), suggesting that there may be two distinct coding regimes.

**Figure 3:**
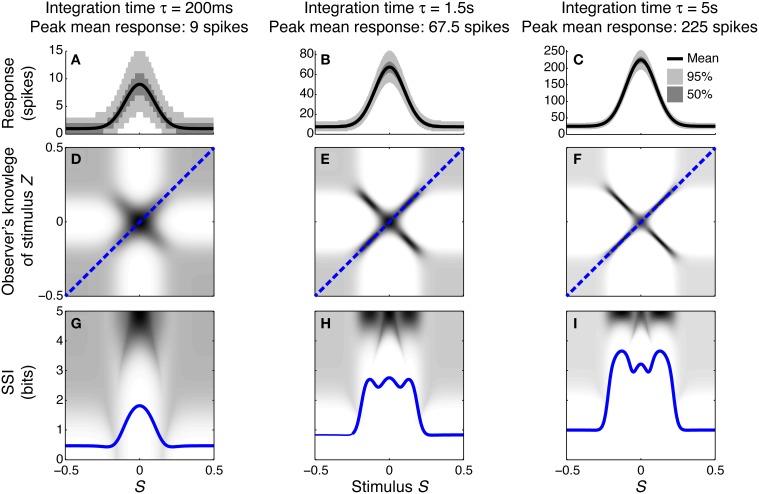
**Bayesian reconstruction with a unimodal tuning curve**. **(A–C)** Show tuning curves and trial-to-trial variability for a single neuron at three different integration times and hence three different levels of trial-to-trial variability. Variability is illustrated by shading: the dark region contains half of the probability mass (25th to 75th percentile), while the light region extends down to the 2.5th percentile and up to the 97.5th percentile and, together with the dark region, contains 95% of the probability mass. **(D–F)** Show *P*(*Z*|*S*), the average posterior stimulus distribution after observing the response to a single trial, conditioned on the true stimulus, for the tuning properties shown in **(A–C)**. Darker shading indicates higher probability and, for clarity, the shading density is scaled independently for each panel and does not span the full [0, 1] interval. This distribution can be read like a lookup table: if the true stimulus is selected on the horizontal axis then the average posterior distribution is given by that column. The more information conveyed by the neuron, the more precise the reconstruction and hence the more the response probability mass is concentrated close to the blue dashed line that corresponds to exact reconstruction (*Z* = *S*). **(G–I)** The SSI for the same three cases overlaid upon the distributions shown in **(D–F)**, where each column of the distribution is sorted so that the highest probability bins are uppermost. This illustrates more clearly the relationship between the SSI and the amount of uncertainty [i.e., the entropy *H*(*Z*|*S* = *s*)] in the distribution *P*(*Z*|*S*); greater SSI corresponds to more precise reconstruction and hence lower posterior entropy. The transition between peak coding and flank coding, according to the SSI, occurs close to the case shown in **(B,E,H)** where the uncertainty associated with the low-gradient peak region of the tuning curve is equal to that due to ambiguity between the two symmetric flanks. Parameters: *N* = 1, *f*_*mod*_ = 40 spikes/s, *f*_*bg*_ = 5 spikes/s, ω = 0.1, σ = 0, stimulus ensemble consists of 401 equally probable discrete stimuli regularly spaced in the interval [−0.5, 0.5].

To illustrate how and why the best-encoded stimuli predicted by the SSI change with the trial-to-trial variability, we simulated a single unimodally-tuned neuron and manipulated the level of variability by changing the integration time. Under the Poisson variability model, as time passes following the presentation of the stimulus the mean spike count increases in proportion to the elapsed time τ, but the standard deviation only increases as $\sqrt{\tau }$, so the signal to noise ratio also increases in proportion to $\sqrt{\tau }$ (Figures 3A–C). Figures 3D–F show the expected posterior distribution on the stimulus conditioned on the true stimulus (*P*(*Z*|*S*); we use the variable *Z* to represent the observer's knowledge of the stimulus to avoid confusion with the true stimulus *S*; see Materials and Methods for further details). This posterior distribution describes the knowledge that a hypothetical observer (e.g., a downstream neuron) has, on average, about the stimulus after receiving the output from our model neuron in a single trial. The symmetric cross shape of the distribution is due to the symmetry of the unimodal tuning curve: because of the ambiguity between the two halves of the tuning curve, it is not possible to distinguish stimuli on one side of the peak from those on the other based on the responses of that neuron alone. In the context of a population, however, the ambiguity between tuning curve flanks tends to be resolved by the responses of the other neurons. Although the gradient of the tuning curve is greatest on its flanks, the inter-flank ambiguity greatly increases the conditional entropy of the posterior. While the distribution is visibly more concentrated around *Z* = *S* and *Z* = −*S* (i.e., precisely correct decoding) when the variability is lower, there is no obvious qualitative change in the distribution between Figures 3D,F that accounts for the change in best-encoded stimulus. The reason for the difference in best-encoded stimuli becomes clearer when each column of the distribution is sorted so that the amount of uncertainty [i.e., the conditional entropy *H*(*Z*|*S* = *s*)] can be seen more easily (Figures 3G,H). Through this visualization, we can see that the transition between peak and flank coding occurs where the uncertainty associated with the two flanks is equal to that of the flat, but narrow, peak (Figures 3B,E,H).

The peak of a tuning curve is associated with a uniquely high mean response, which is more different from the background response than any other point on the tuning curve. Responses to stimuli around the peak are therefore always informative as they allow coarse discrimination of the peak from untuned background activity, and this contribution to the overall information dominates when the variability is high (e.g., Figures 3A,D,G). In order to discriminate between closely neighboring stimuli and hence estimate the stimulus more precisely, the responses associated with those stimuli must be different. This fine discriminability is quantified by the Fisher information and is maximal where the gradient of the tuning curve is greatest. When the variability is low, fine discrimination dominates, with the result that the best-encoded stimulus shifts to the flanks of the tuning curve (Butts and Goldman, 2006, and Figures 3C,F,I).

#### 3.1.2. Monotonic tuning curves

**Figure 4:**
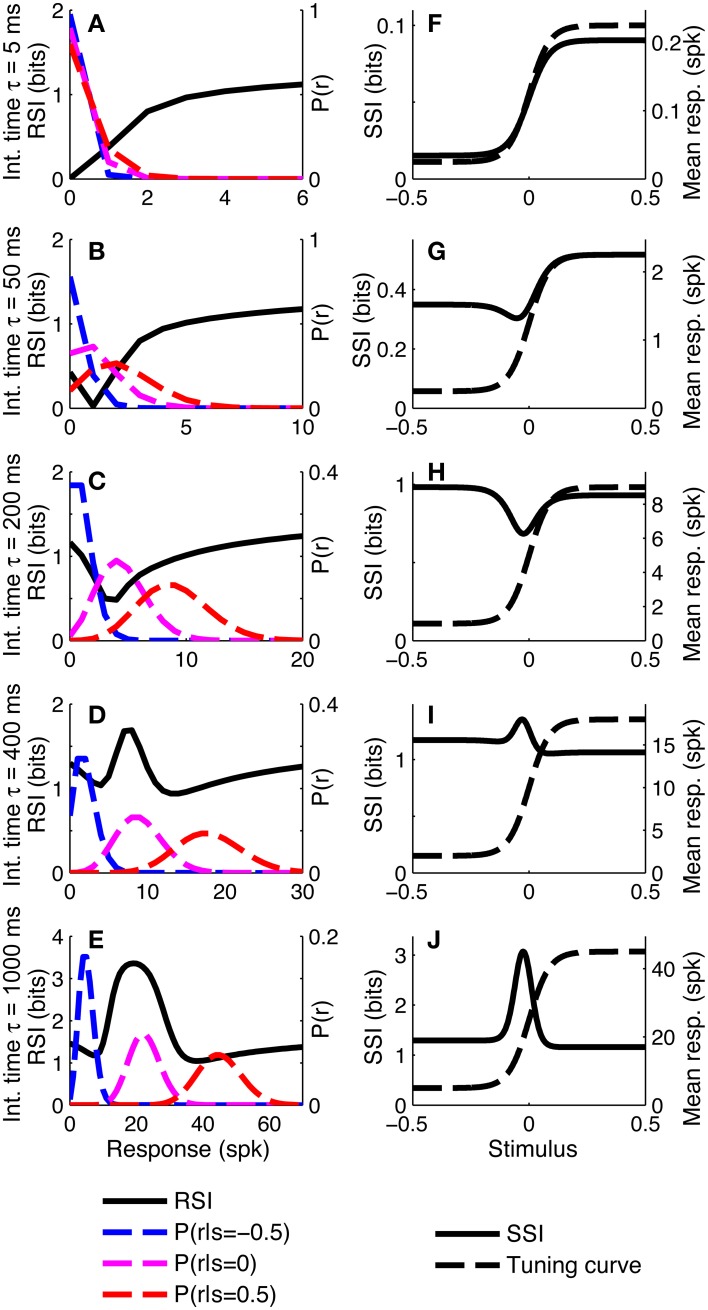
**SSI for a sigmoidal monotonic tuning curve**. **(A–E)** show the response specific information (RSI), together with response distributions for low, mid and high responding regions of the tuning curve. These are shown for five different levels of trial-to-trial variability determined by the integration time τ; short integration times lead to high variability and vice versa. The differences in trial-to-trial variability are best illustrated by the increasing separation between the high, mid and low response distributions. **(F–J)** give the SSI for the same five sets of parameter values. Parameters: *N* = 1, *f*_*mod*_ = 40 spikes/s, *f*_*bg*_ = 5 spikes/s, ω = 0.044, σ = 0, stimulus ensemble consists of 401 equally probable discrete stimuli regularly spaced in the interval [−0.5, 0.5].

**Figure 5:**
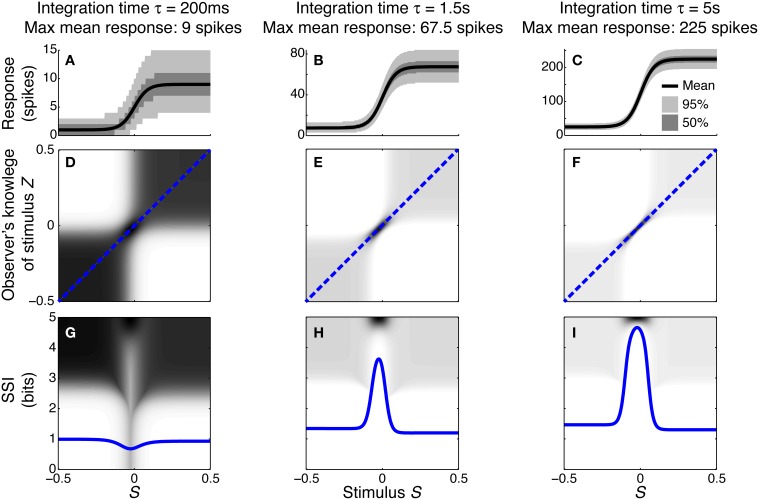
**Bayesian reconstruction with a monotonic tuning curve**. **(A–C)** tuning curves and trial-to-trial variability for a single neuron at three different integration times and hence three different levels of trial-to-trial variability. Variability is illustrated by shading: the dark region contains half of the probability mass (25th to 75th percentile), while the light region extends down to the 2.5th percentile and up to the 97.5th percentile and, together with the dark region, contains 95% of the probability mass. **(D–F)** *P*(*Z*|*S*), the average posterior stimulus distribution after observing the response to a single trial, conditioned on the true stimulus, for the tuning properties shown in **(A–C)**. Darker shading indicates higher probability and, for clarity, the shading density is scaled independently for each panel and does not span the full [0, 1] interval. This distribution can be read like a lookup table: if the true stimulus is selected on the horizontal axis then the average posterior distribution is given by that column. The more information conveyed by the neuron, the more precise the reconstruction and hence the more the response probability mass is concentrated close to the blue dashed line that corresponds to exact reconstruction (*Z* = *S*). **(G–I)** the SSI for the same three cases overlaid upon the distributions shown in **(D–F)**, where each column of the distribution is sorted so that the highest probability bins are uppermost. This illustrates more clearly the relationship between the SSI and the amount of uncertainty (i.e., the entropy *H*(*Z*|*S* = *s*)) in the distribution *P*(*Z*|*S*); greater SSI corresponds to more precise reconstruction and hence lower posterior entropy. Two contrasting coding regimes can be seen: a Fisher-like regime (best-encoded stimulus at the steepest part of the tuning curve **(H,I)**, and a regime where the SSI is relatively flat, with minimum information occurring at the steep region of the tuning curve **(G)**. Parameters: *N* = 1, *f*_*mod*_ = 40 spikes/s, *f*_*bg*_ = 5 spikes/s, ω = 0.044, σ = 0, stimulus ensemble consists of 401 equally probable discrete stimuli regularly spaced in the interval [−0.5, 0.5].

To investigate whether similar distinct coding regimes could also exist for monotonic tuning curves, we calculated the SSI for a single sigmoidally-tuned model neuron with Poisson trial-to-trial variability. The tuning curve parameters were fixed and the level of variability was manipulated by changing the integration time. Figure 4 shows the SSI and the RSI for the model neuron at several different integration times from 5 ms to 1 s. At very short integration times (very high trial-to-trial variability) the RSI increases monotonically with increasing spike count (Figure 4A) and the SSI is almost proportional to the tuning curve (Figure 4F). The mean responses to all stimuli are less than one spike, therefore the Poisson response distributions for all stimuli are monotonically decreasing with maxima at zero spikes. At this point in most trials no spikes have occurred yet, regardless of the stimulus, so zero spike counts are uninformative, as indicated by their low RSI. Non-zero responses, however, are more likely to have been caused by stimuli at the high-responding region of the tuning curve (we refer to this as the tuning curve plateau), so these have higher RSI. As the integration time increases and the mean responses to plateau stimuli increase above one spike (Figures 4B,C), these response distributions are no longer monotonic, but peaked around the expected response. This means that very low spike counts are indicative of stimuli on the non-selective, low-responding “baseline” region of the tuning curve, while high responses are more likely to be caused by stimuli on the plateau. Both high and very low spike counts therefore have relatively high RSI, and there is a trough in the RSI curve at intermediate responses (Figures 4B,C) and a corresponding trough in the SSI at the flank (sloping region) of the tuning curve (Figures 4G,H). This is the coarse discrimination regime for monotonic tuning curves, analogous to the peak coding regime for unimodal tuning curves. Here the neuronal responses support discrimination of baseline from plateau stimuli, but are too noisy to allow neighboring stimuli to be distinguished from one another. This can be seen in the conditional posterior distribution *P*(*Z*|*S*); the chequerboard pattern in Figure 5D means that stimuli can be decoded as being from either the baseline or plateau regions of the tuning curve, but stimuli from within either region are indistinguishable. The distributions of responses to stimuli on the flank of the tuning curve overlap with those of the baseline and plateau, so these intermediate responses could have been caused by any stimulus and are therefore uninformative. This is clearly visible as the dark central band in Figure 5G, which shows that the posterior distribution for stimuli on the flank of the tuning curve is broad rather than tightly peaked around the correct stimuli. Within the coarse discrimination regime the relative values of the SSI for the baseline and plateau regions are largely determined by the characteristic stimulus of the neuron: the position of the tuning curve flank within the stimulus space. If the plateau is relatively narrow then high spike counts identify a smaller range of stimuli than low spike counts and the SSI is at its greatest on the plateau. Conversely, and perhaps unintuitively, if the plateau is broader than the baseline region then low spike counts are more informative and the maximum SSI occurs on the baseline region (results not shown). When the flank is located centrally in the stimulus space, the SSI of the plateau and baseline regions are similar, as in Figure 4H.

As the integration time is increased and the trial-to-trial variability consequently decreases, the RSI for intermediate spike counts (Figure 4D) and the SSI at the flank of the tuning curve (Figure 4I), increase and become maxima (both were minima at shorter integration times). The best-encoded stimulus is now at the flank of the tuning curve. This is the emergence of the fine discrimination regime; here the trial-to-trial variability is sufficiently low to allow discrimination between stimuli on the flank of the tuning curve. The gradient of the tuning curve is greatest in the center of the flank, and it is here that the Fisher information is greatest and the response distributions of adjacent stimuli are most different. The relatively high precision of decoding on the flank can be seen in the conditional posterior distributions (Figures 5E,H) as a concentration of posterior probability mass around the correct stimulus (*Z* = *S*). Further increases in integration time increase the maximum values of the RSI (Figure 4E) and SSI (Figure 4J; see also Figures 5F,I), but the best-encoded stimulus remains the same.

#### 3.1.3. Summary

Monotonic tuning curves exhibit distinct coding regimes analogous to the peak and flank regimes of unimodal tuning curves. When the trial-to-trial variability is low, fine discrimination dominates and the maximum SSI is on the flank of the tuning curve, where the Fisher information is also maximal, and hence both measures predict similar best-encoded stimuli. For high variability, coarse discrimination dominates and the best encoded stimulus is less easy to predict. Monotonic tuning curves differ from unimodal tuning curves in that the high-response region is broad rather than localized. This means that, as described above, strong responses are not always informative (as is the case for peaked tuning curves), as any stimulus on the upper plateau of the tuning curve is likely to generate a strong response. The best-encoded stimulus for a single monotonically-tuned neuron is not clearly defined, as maximum SSI can occur over the whole of the plateau or the whole of the baseline region. Another important difference between monotonic and unimodal tuning curves is symmetry: as monotonic tuning curves have only a single flank, each stimulus on the flank is unambiguously associated with a unique response distribution. This means that, for a given tuning curve gradient and variability (equal Fisher information), responses to stimuli on the tuning curve flank are more informative than they would be for a single neuron with a unimodal tuning curve. For example, compare the SSI peaks close around the threshold stimulus in Figures 5H,I (approximately 3.5 and 4.5 bits, respectively) with the SSI peaks at the tuning curve flanks in Figures 3H,I (approximately 2.5 and 3.5 bits).

### 3.2. The effect of population size

Population size is an important determinant of the magnitude and shape of the SSI for population coding neurons, and hence of the best-encoded stimulus. To quantify the information contributed by a neuron in the context of a population code, we use the marginal SSI (mSSI). The mSSI of a neuron is the difference between the SSI of the entire population and the SSI of the population without the neuron of interest. It is useful to think of the single-neuron SSI and marginal SSI as upper and lower bounds, respectively, on the informational contribution of a neuron to a population code. One way to understand this is to imagine building up a population by adding one neuron at a time. Assuming that the code is redundant (as population codes of the type modeled here are), each successive neuron will result in a smaller and smaller additions to the population SSI: the first increment is the single-neuron SSI and the last is the marginal SSI.

#### 3.2.1. Unimodal tuning curves

Previous research on unimodal tuning curves showed that increasing the number of neurons in the population shifts the code toward the asymptotic regime, where Fisher information accurately predicts both the mutual information between stimulus and response (Brunel and Nadal, 1998), and the best-encoded stimulus predicted by the mSSI (Yarrow et al., 2012). In large populations the best-encoded stimuli predicted by the mSSI lie on the flanks of the tuning curves and coincide with the Fisher information maxima. In small populations with high trial-to-trial variability, the best-encoded stimuli according to the mSSI are at the peaks of the tuning curves. The number of neurons at which the transition between the peak and flank coding regimes occurs depends upon the variability: if the variability is low, each neuron conveys a correspondingly higher amount of information and the peak to flank transition occurs at relatively low *N*. The higher the variability, the larger the population must be before it enters the asymptotic regime. Other factors, including the strength and structure of correlations in the trial-to-trial variability, also affect the population size at which the asymptotic regime is reached, but these effects are relatively small (see Yarrow et al., 2012, for details).

#### 3.2.2. Monotonic tuning curves

**Figure 6:**
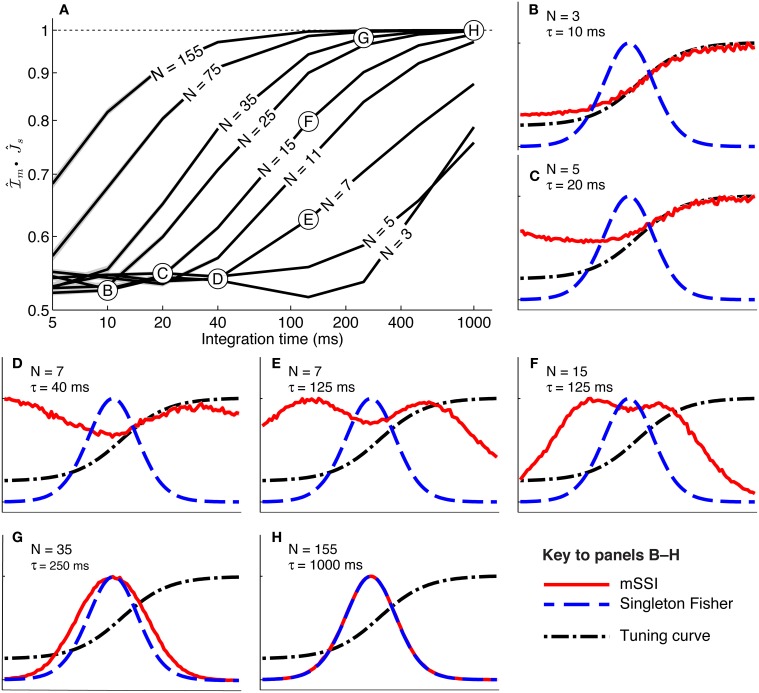
**Marginal SSI in populations of sigmoidally tuned neurons**. **(A)** The normalized dot product of the mSSI and the singleton Fisher information (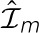 • ${\hat{J}}_{s}$) as a function of integration time τ for various population sizes *N*. This indicates the similarity in shape of the mSSI and the Fisher information; a value of 1 indicates that the mSSI is directly proportional to the Fisher information, while low values indicate that the two measures have dissimilar shapes. **(B–H)** mSSI as a function of the stimulus for parameter values corresponding to the points labeled in **(A)**. The Fisher information and tuning curve for the neuron of interest are also shown. All quantities in **(B–H)** are normalized. Parameters: *f*_*mod*_ = 40 spikes/s, *f*_*bg*_ = 10 spikes/s, ω = 0.1, σ = 0, stimulus variable is continuous in the interval [−1, 1].

To study how population size affects the best-encoded stimulus for monotonic tuning curves, we calculated the mSSI for populations of *N* model neurons with stereotypical sigmoidal tuning curves and Poisson trial-to-trial variability (again controlled by the integration time τ). Our results show that, as for unimodal tuning curves, the shape of the mSSI converges toward the shape of the single-neuron Fisher information as the number of neurons in the population increases (Figure 6A). In the asymptotic regime, the best-encoded stimulus is on the flank of the tuning curve (Figure 6H). The rate with which the shape of the mSSI converges to the shape of the Fisher information with increasing population size depends on the level of variability; as for unimodal tuning curves, the higher the variability, the slower the convergence and the larger the population at which the asymptotic regime is reached (Figure 6A). Like the SSI for single neurons, the shape of the mSSI in small populations with high variability is close to that of the tuning curve (Figure 6B) and the best-encoded stimuli are those on the tuning curve plateau.

Between these extremes of a small population with high variability and a large population with low variability, the mSSI for a unimodal tuning curve can take on variety of different shapes and consequently the best-encoded stimuli vary widely. The mSSI goes through a predictable progression of shapes as the trial-to-trial variability is decreased (by increasing the integration time), or the population size increased. As we have already seen, the mSSI is sigmoidal for small populations and short integration time (Figure 6B). If τ or *N* is increased slightly, the mSSI for the baseline region of the tuning curve increases and an mSSI peak forms at the lower end of the tuning curve flank (Figures 6C–E). This peak in the mSSI becomes sharper and moves toward the the characteristic stimulus (the center of the tuning curve flank) as τ or *N* increase (Figures 6D,F). At the same time, the mSSI around the tuning curve plateau changes from the flat upper part of a sigmoid into a second peak, which also becomes sharper and migrates toward the characteristic stimulus as τ or *N* is increased (Figures 6C–F). At these intermediate, transitional values of τ and *N*, the mSSI is bimodal, with both peaks having approximately equal magnitude. As τ or *N* is increased further, the two mSSI peaks ultimately merge into a single peak located at the tuning curve flank, coincident with the maximum Fisher information (Figures 6E–G). This convergence of the best-encoded stimuli predicted by the mSSI and the Fisher information occurs at population sizes that are modest in the context of the mammalian brain: the shapes of the two measures are close even in populations of the order of 100 neurons at biologically relevant integration times (of the order of 100 ms; Figure 6A).

#### 3.2.3. Summary

The single neuron SSI for monotonically-tuned neurons is dependent upon the level of trial-to-trial variability and the marginal SSI is dependent upon the variability and the population size. For large populations the best-encoded stimulus lies on the sloping region of the tuning curve, close to the point of maximum gradient, as predicted by the Fisher information. In smaller populations the best-encoded stimulus is dependent on the variability and can be anywhere on, or close to, the sloping part of the tuning curve. These findings are in agreement with those for unimodal tuning curves (Butts and Goldman, 2006; Yarrow et al., 2012), but the shape of the SSI—and hence the best-encoded stimulus—is more varied in the case of monotonic tuning curves.

### 3.3. Non-uniform stimulus distributions

**Figure 7:**
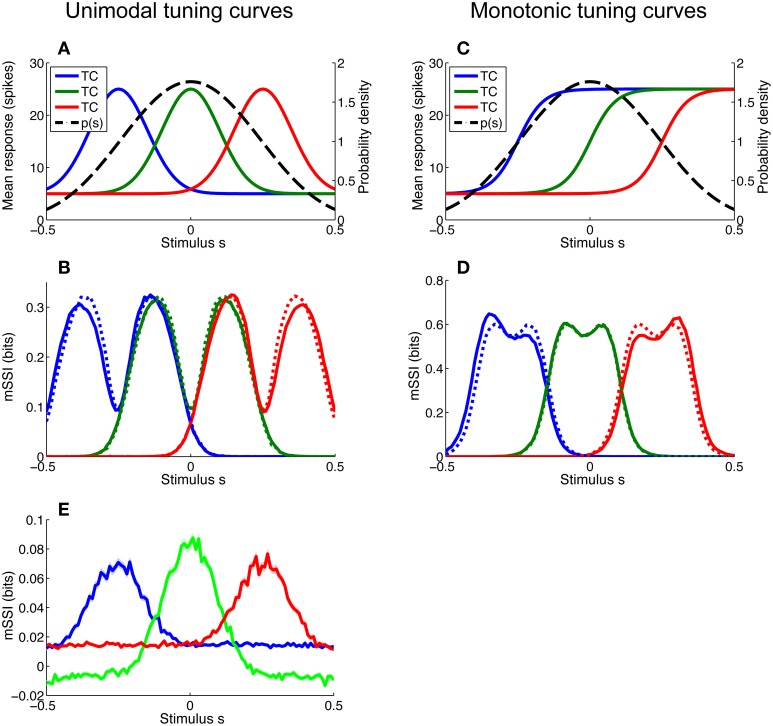
**Effect of stimulus distribution non-uniformity on the marginal SSI**. Non-uniformity of the stimulus distribution can affect the marginal SSI. Here we consider a stimulus distribution that is peaked around zero and compute the mSSI for three neurons, one at the peak of the stimulus distribution and one on either flank. **(A)** Stimulus distribution *p*(*s*) and tuning curves (TC) for unimodal neurons of interest. **(B)** mSSI for the tuning curves and stimulus distribution shown in A (solid lines), together with the mSSI for identical tuning curves and variability, but a uniform stimulus distribution (broken lines). **(C–D)** As **(A–B)**, for monotonic tuning curves. **(E)** The mSSI for the same neurons illustrated in **(A)**, but with a very short integration time (τ = 5 ms). Notice the negative mSSI values coinciding with the tails of the central (green) tuning curve. Uncertainty in the mSSI is indicated by shaded regions of ± 1 Standard Error where it is greater than the width of the line. Parameters: *N* = 17, τ = 500 ms, *f*_*mod*_ = 40 spikes/s, *f*_*bg*_ = 10 spikes/s, ω = 0.1 (unimodal), ω = 0.044 (monotonic), stimulus variable is continuous and distributed as shown, or uniformly across the interval [−1, 1].

For most of the simulations described in this article we have assumed that stimuli are uniformly distributed, but this is rarely the case in nature. Even stimulus variables that seem uniformly distributed may not be; for example edge orientation in natural scenes is non-uniformly distributed, with horizontal and vertical contours occurring more frequently than other orientations (Coppola et al., 1998). In particular, monotonic tuning curves imply a linear (as opposed to circular) stimulus space and this, in turn, implies that the stimuli are non-uniformly distributed such that they fall within some finite interval; a uniform distribution within sharply defined limits is extremely unlikely. As the space of possible stimulus distributions is essentially limitless, we did not attempt to examine a wide variety of distribution forms and instead focussed on determining the effect of local non-uniformity of the stimulus distribution on the mSSI of neurons with both unimodal and monotonic tuning curves. To this end, we modeled a broadly peaked stimulus distribution *p*(*s*) and considered three neurons of each tuning curve type: one with its characteristic stimulus at the peak of the stimulus distribution and one on either flank (see Figures 7A,C). The mSSI of each of the neurons of interest was computed for both the peaked stimulus distribution and a uniform distribution extending well beyond the tuned regions of the neurons (Figures 7B,D). The simulations were repeated for a range of population sizes and integration times.

As previously discussed, the mSSI peaks converge toward those of the Fisher information as the population size goes to infinity (Yarrow et al., 2012). This holds for non-uniform stimulus distributions, so the stimulus distribution (provided that it is non-zero) has no effect upon the best-encoded stimulus predicted by the mSSI in the limit of large populations (this was confirmed by numerical simulation; results not shown). As we shall see, away from the asymptotic regime the stimulus distribution can strongly affect the best-encoded stimulus, particularly for neurons with monotonic tuning curves.

#### 3.3.1. Unimodal tuning curves

For small populations and high variability (i.e., in the peak coding regime) the peak of a unimodal tuning curve defines the best-encoded stimulus, and the stimulus distribution has little or no effect. Therefore, non-uniformity of the stimulus distribution affects the best-encoded stimulus primarily in the transition between the peak and flank coding regimes, where the mSSI is bimodal (as in Figure 7B). The effect of non-uniformity is to skew the mSSI toward greater stimulus probability: the mSSI peak coinciding with the greater stimulus probability is amplified, while the other is suppressed. Thus, the best-encoded stimulus is shifted toward the peak of the stimulus distribution.

#### 3.3.2. Monotonic tuning curves

The best-encoded stimuli of monotonically-tuned neurons are more strongly affected by the stimulus distribution, and the effect is greatest for small populations with high variability (results not shown). As is the case for the single-neuron SSI, it is difficult to predict the shape of the mSSI under these conditions without actually computing it, as it is sensitive to the tuning curve shape, the position of the characteristic stimulus within the stimulus range, as well as to the stimulus distribution. For monotonic tuning curves, the effect of non-uniformity in the stimulus distribution is that the mSSI peak with lower stimulus probability is amplified and the peak with higher stimulus probability is attenuated (Figure 7D); thus the best-encoded stimulus is shifted away from the most probable stimulus. This is the opposite of the effect that occurs with peaked tuning curves.

#### 3.3.3. Unexpected stimuli

The presence of unexpected stimuli—those with a low probability of occurrence—in a stimulus ensemble can lead to counterintuitive SSI values. If much of the probability mass in the stimulus distribution is concentrated around a subset of common stimuli, then evidence of an unusual stimulus causes an initial increase in the posterior entropy; this can be seen as a negative SSI or mSSI (Figure 7E shows an example of this: the mSSI at the tails of the central (green) tuning curve are slightly negative). Although the SSI is transiently negative, the weak evidence provided by the early response does make the posterior more “correct” i.e., it increases the posterior probability of the true stimulus relative to other stimuli. This odd property of the SSI serves as a reminder that care is sometimes required when interpreting information theoretic measures. It is important to note that a locally negative SSI does not violate the non-negativity of Shannon information, as the mutual information—the expected value of the SSI—remains non-negative.

#### 3.3.4. Summary

The stimulus distribution itself can affect the shape of the mSSI and the best-encoded stimulus in the pre-asymptotic regime. When there is a gradient in the stimulus distribution *p*(*s*) around the characteristic stimulus of a neuron (peak of unimodal, or flank of monotonic tuning curve), the best-encoded stimulus is shifted in the direction of greater *p*(*s*) in the case of unimodal tuning curves and in the opposite direction in the case of monotonic tuning curves. In small, noisy populations with monotonic tuning curves the stimulus distribution can strongly effect the best-encoded stimulus; such cases should be analyzed individually.

### 3.4. Best-encoded stimuli in forced choice tasks

The preceding sections described how the best-encoded stimulus of a neuron depends upon trial-to-trial variability and population size when there are many possible stimuli. Large changes in the best-encoded stimulus, such as the peak-flank transition for unimodal tuning curves, are caused by changes in the relative amounts of information associated with fine vs. coarse discrimination (Butts and Goldman, 2006). These effects can only be observed when the stimulus ensemble is sufficiently rich, in the sense that it must include both narrowly and widely separated stimuli so that both fine and coarse discrimination are relevant. When applied to rich stimulus ensembles, information theoretic measures, such as the SSI, quantify information in a way that is not specific to any particular behavioral task. Sometimes, however, it is useful to predict what the best-encoded stimulus is for a specific behavioral task, particularly when comparing precision at the neural and behavioral levels, as behavioral experiments typically involve simplified tasks (e.g., two-alternative forced choice; 2AFC). This type of task involves making a decision between predefined choices, and the SSI can be used to analyze these tasks by tailoring the stimulus ensemble to match the choices in the task: for instance, a 2AFC task corresponds to an ensemble with two stimuli. Modeling the task in this way allows us to identify the stimulus values for which a neuron conveys the most decision-relevant information, where the decision to be made is defined by the task.

#### 3.4.1. Unimodal tuning curves

In single neurons with unimodal tuning curves, when the stimulus ensemble is simple (contains few stimuli) it is the ensemble itself that determines the best-encoded stimulus (Butts and Goldman, 2006). For example, in a 2AFC task involving two closely-spaced stimuli (i.e., when Δ*s* is much less than the width of the tuning curve flank), coarse discrimination is irrelevant as there are no widely-spaced stimuli in the ensemble, so information about the stimulus is maximized when the stimuli fall on the steepest part of the tuning curve flank, where Fisher information is maximal. Thus, for closely-spaced stimuli the best-encoded stimuli are on the flanks of the tuning curve, meaning that a neuron conveys the most information in support of a decision between the two stimuli when they fall on a flank of the tuning curve. Conversely, for widely spaced pairs of stimuli (such that when one lies at the tuning curve peak the other lies on the non-tuned baseline region) fine discrimination is irrelevant and the best-encoded stimulus is at the peak of the tuning curve. Because the coding regime is determined entirely by the stimulus ensemble, the best-encoded stimulus is not affected by the level of trial-to-trial variability as they are when the stimulus ensemble is rich.

**Figure 8:**
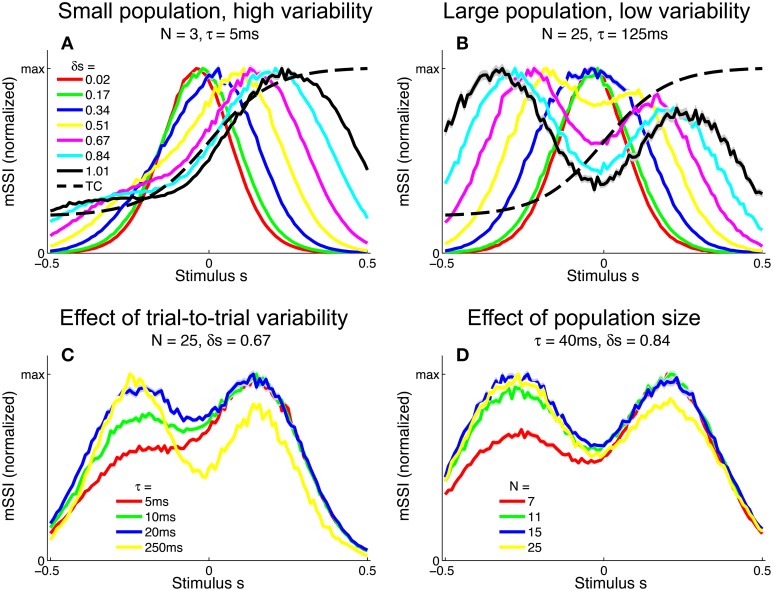
**Best-encoded stimuli for unimodal tuning curves in a two-alternative discrimination task**. **(A)** mSSI of a neuron in a very small population with high trial-to-trial variability, for two-alternative discrimination tasks with varying normalized stimulus interval δ*s*. **(B)** As **(A)**, but for a slightly larger population and lower variability. **(C–D)** Show typical effects of changing the variability and population size, respectively. Uncertainty in the mSSI is indicated by shaded regions of ± 1 Standard Error where it is greater than the width of the line. Parameters: *f*_*mod*_ = 40 spikes/s, *f*_*bg*_ = 10 spikes/s, ω = 0.1.

To investigate the effect of the task (stimulus ensemble) on the best-encoded stimuli in the context of a population code, we computed the mSSI for unimodal tuning curves in 2AFC tasks with normalized stimulus spacings in the range δ*s* = [0.1, 3.5]. Our results show that the best-encoded stimuli indicated by the mSSI in 2AFC tasks is determined by the stimulus spacing in the same way that the single-neuron SSI is, i.e., it is at the tuning curve peak for coarse discrimination and at the flanks for fine discrimination (see Figures 8A,B; the transition between coarse and fine discrimination occurs at approximately δ*s* = 1). With unimodal tuning curves, the trial-to-trial variability and the size of the population have little effect upon the best-encoded stimulus. In the coarse discrimination regime, the only effect of changing the level of variability (Figure 8C) or the number of neurons in the population (Figure 8D) is to change the height of the secondary mSSI peaks relative to the central maximum. In the fine discrimination regime, neither the level of variability or the population size have any effect on best-encoded stimulus—it is always the same as that predicted by the Fisher information, but for intermediate, transitional stimulus spacings where the mSSI is bimodal, changes in τ can cause small shifts in the best-encoded stimuli (results not shown).

#### 3.4.2. Monotonic tuning curves

**Figure 9:**
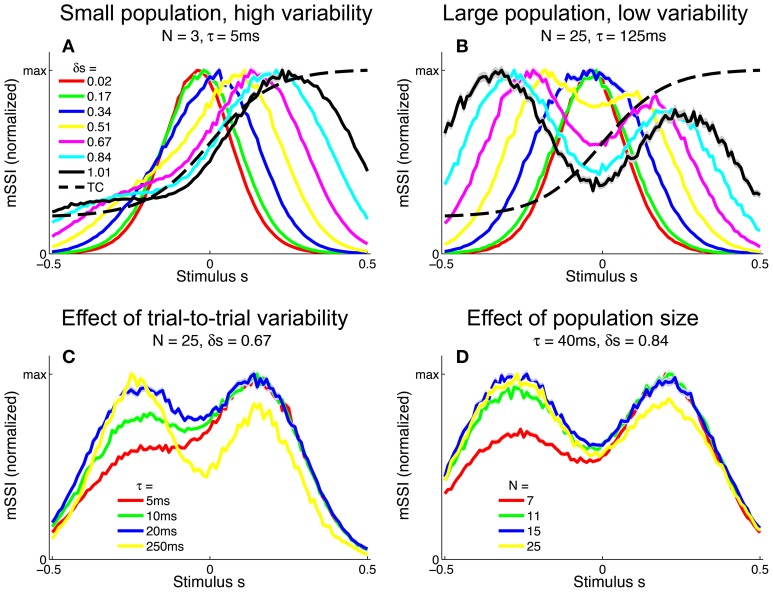
**Best-encoded stimuli for monotonic tuning curves in a two-alternative discrimination task**. **(A)** mSSI of a neuron in a very small population with high trial-to-trial variability, for two-alternative discrimination tasks with varying normalized stimulus interval δ*s*. **(B)** As **(A)**, but for a slightly larger population and lower variability. **(C,D** Show typical effects of changing the variability and population size, respectively. Uncertainty in the mSSI is indicated by shaded regions of ± 1 Standard Error where it is greater than the width of the line. Parameters: *f*_*mod*_ = 40 spikes/s, *f*_*bg*_ = 10 spikes/s, ω = 0.1.

We extended the analysis to populations of monotonic tuning curves by again computing the mSSI for 2AFC tasks with stimulus spacings in the range δ*s* = [0.01, 1]. For monotonic tuning curves, the best-encoded stimulus for fine discrimination tasks is again at the point of maximum Fisher information i.e., the flank of the tuning curve (Figure 9). However, for coarse discrimination tasks, predicting the best-encoded stimulus is less straightforward than is the case for peaked tuning curves, as the shape of the mSSI is somewhat dependent on trial-to-trial variability and population size. As the stimulus interval is increased, the best encoded stimulus shifts toward the upper end of the tuning curve flank (Figure 9A). For coarse discrimination a secondary mSSI peak emerges on the low-responding side of the flank and grows as the variability is reduced or the population size increased (Figure 9B). The two peaks are due to the two components of the SSI in a two-alternative task (see Materials and Methods), each of which is bell-shaped. The distance between the peaks of the two components is determined by the stimulus interval, until the stimulus interval is greater than the width of the tuning curve flank. The effect of decreasing the variability (Figure 9C) or increasing the number of neurons in the population (Figure 9D) is to increase the magnitude of the mSSI peak on the low-responding side of the tuning curve; eventually this peak exceeds the one at the plateau end of the flank, and the best-encoded stimulus shifts to the baseline end of the flank. In general, the best-encoded stimulus is at the baseline end of the tuning curve flank for large populations, low variability or widely-spaced stimuli.

#### 3.4.3. What constitutes coarse discrimination?

In contrast to the SSI, the Fisher information is specific to tasks where information on fine-grained stimulus differences dominates—fine discrimination, estimation, reconstruction—and does not quantify information that contributes to coarse discrimination. The Fisher information always predicts that the best-encoded stimuli coincide with the flanks of peaked tuning curves, and when the SSI is used to analyze equivalent fine-grained tasks it yields similar predictions. So far, we have described the stimulus spacing in 2AFC tasks rather loosely as being coarse or fine, but what actually constitutes a “fine” or “coarse” discrimination task? Where is the boundary between fine and course discrimination, and what are the limits of applicability of Fisher information in terms of stimulus spacing; when does Fisher information correctly identify the best-encoded stimulus? What if there are more than two alternatives in a forced choice task?

**Figure 10:**
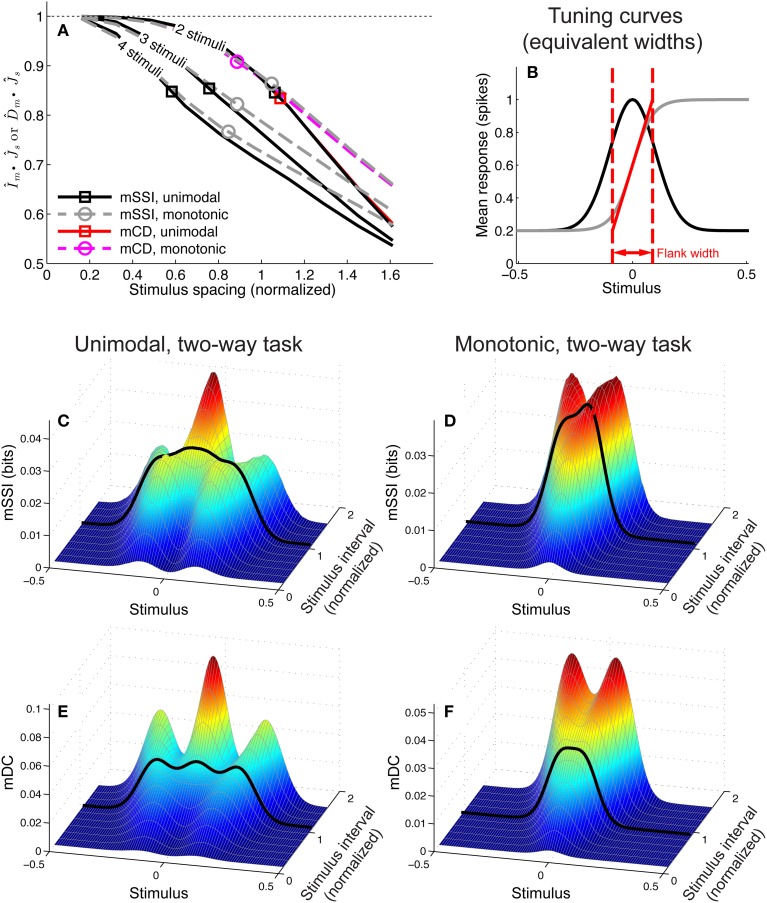
**Best-encoded stimulus depends on stimulus spacing in a forced-choice task**. This figure summarizes how the best-encoded stimulus predicted by the marginal SSI and marginal Chernoff distance diverges from that predicted by the Fisher information as the distance between stimuli is increased in a forced-choice discrimination task. **(A)** Normalized dot product (see Materials and Methods) of mSSI and Fisher information (for 2, 3, and 4-alternative tasks) together with the normalized dot product of Chernoff distance and Fisher information (for 2-alternative tasks), for unimodal and monotonic tuning curves. Markers indicate the approximate transition point between the fine and coarse discrimination regimes. The interval between stimuli δ*s* is normalized such that it is expressed as a fraction of the tuning curve flank width as shown in **(B)** (see also Materials and Methods). **(B)** Tuning curves; the widths of unimodal and monotonic tuning curves were chosen such that both have equal peak Fisher information, equal maximum gradient, and equal flank width (see Materials and Methods). **(C)** mSSI of a neuron in a population of 81 with unimodal tuning curves, for 2-alternative forced choice tasks with normalized stimulus intervals ranging from δ*s* = 0.16 to δ*s* = 1.6. The heavy black line indicates the approximate transition point as shown in **(A)**. **(D)** As **(C)**, for monotonic tuning curves. **(E,F)** As **(C–D)**, but showing the marginal Chernoff distance. Parameters: *N* = 81, τ = 20 ms, *f*_*mod*_ = 40 spikes/s, *f*_*bg*_ = 10 spikes/s, ω = 0.1 (unimodal), ω = 0.044 (monotonic).

To address these questions we computed the mSSI for two, three and four-alternative forced choice tasks with a range of stimulus spacings, for populations of 81 neurons with either unimodal or monotonic tuning curves (Figure 10). We modeled normalized stimulus spacings in the range δ*s* = [0.16, 1.6], which covers the transition between the fine and coarse discrimination regimes. For both types of tuning curve, the transition between fine discrimination and coarse discrimination, according to the mSSI, occurs at approximately δ*s* = 1 (Figure 10A), i.e., where the distance between the stimuli is roughly the same as the width of the tuning curve flank (Figure 10B). Increasing the number of alternatives in the task introduces elements of coarser discrimination; for example, a three-alternative task involves distinguishing between non-adjacent stimuli separated by 2 Δ*s*, as well as adjacent stimuli with an interval of Δ*s*. The presence of more widely spaced stimulus pairs within the ensemble can drive the shape of the mSSI toward the coarse discrimination regime i.e., a shape less like that of the Fisher information, as indicated by lower ${\hat{I}}_{m}•\hat{J}$ values (Figure 10A; see Materials and Methods for more details of how we quantify similarity of shape). Figure 10C shows how the mSSI varies with δ*s* for a two-alternative task and unimodal tuning curves; the transition between the Fisher-like bimodal shape of the fine discrimination regime and the strong central peak of the coarse discrimination regime can be clearly seen. Similarly, for monotonic tuning curves, the transition is between unimodal mSSI for fine discrimination (best-encoded stimulus at the flank of the tuning curve) and bimodal mSSI for coarse discrimination (best-encoded stimulus near one end of the flank), as can be seen in Figure 10D.

#### 3.4.4. Chernoff distance: an efficient measure for 2AFC tasks

For two-alternative tasks, the Chernoff distance can be used to measure the discriminability of the two stimuli. To compare the best-encoded stimuli predicted by the SSI and Chernoff distance, we computed the marginal Chernoff distance (mDC; see Materials and Methods) for the same population models and tasks described in the preceding paragraph (Figures 10E,F). For unimodal tuning curves, the difference in shape between the mSSI and mDC is primarily in the outer information peaks located at approximately ± Δ*s* (Figures 10C,E), and this has little effect on the best-encoded stimulus. The asymmetry of the monotonic tuning curve, however, reveals an important difference between the mSSI and mDC: because the Chernoff distance is commutative [*D*(*s*, *s* + Δ*s*) = *D*(*s* + Δ*s*, *s*)] both components of the mDC are identical, but shifted by Δ*s*. This means that the mDC for the monotonic tuning curve is symmetrical, with two information peaks of equal height and thus two best-encoded stimuli. Although the shapes of the mSSI and mDC are not exactly the same, the best-encoded stimuli predicted by the marginal Chernoff distance are qualitatively consistent with those predicted by the mSSI and, as for the mSSI, the transition between fine and coarse discrimination regimes occurs at approximately δ*s* = 1. The shape of the mDC is also very close to the shape of the Fisher information for fine discrimination: ${\hat{D}}_{m}•\hat{J}$ approaches 1 as δ*s* → 0 (Figure 10A). The best-encoded stimulus predictions of the Chernoff distance agree with those of the SSI, and the marginal Chernoff distance is therefore a computationally efficient method of estimating best-encoded stimuli for two-alternative tasks.

#### 3.4.5. Summary

When the task in hand limits the possible stimulus choices, such as in a 2AFC protocol, the spacing between the alternatives determines the best-encoded stimulus. If the stimulus alternatives are separated by less than the width of the tuning curve flank, a neuron is most informative when the stimuli fall on the steepest parts of the tuning curve flanks, as predicted by the Fisher information. For stimulus spacings greater than the width of the tuning curve flank, the best-encoded stimuli are at the tuning curve peak for unimodal tuning curve, and at either end of the flank for monotonic tuning curves. The Chernoff distance can be used to assess best-encoded stimulus for 2AFC tasks, and is an efficient alternative to the SSI while yielding qualitatively equivalent predictions.

## 4. Discussion

We have shown how the best-encoded stimulus for neurons with sigmoidal monotonic tuning curves depends on the number of neurons in the population, the level of trial-to-trial variability and on the location of the tuning curve flank within the stimulus space. This builds upon earlier studies (Butts and Goldman, 2006; Yarrow et al., 2012) that showed how best-encoded stimuli for unimodal tuning curves depend on the level of trial-to-trial variability and the population size. For large populations (of the order of hundreds of neurons), we confirmed by numerical simulation that the best-encoded stimulus predicted by the SSI for monotonically tuned neurons agrees with that predicted by the Fisher information: i.e., the best-encoded stimulus is on the sloping flank of the tuning curve. This is in agreement with the earlier studies addressing unimodal tuning curves and also with the proven equivalence of Fisher information and mutual information in the limit as the population size *N* → ∞ (Brunel and Nadal, 1998). Away from the asymptotic regime, in smaller populations where the shapes of the marginal SSI and the Fisher information differ, the best-encoded stimulus is harder to predict as it can be strongly affected by the population size, variability and stimulus distribution as well as the tuning curve; this is in contrast to peaked tuning curves, where the SSI is greatest at the peak of the tuning curve under similar conditions. This difference is due to the fact that strong responses by neurons with saturating monotonic tuning curves are often triggered by a wider range of stimuli, which makes them less informative. Far from the asymptotic regime, the best-encoded stimuli can be at either end of the tuning curve flank, and may extend (i.e., the SSI or mSSI may be flat) across either the plateau or baseline regions of the tuning curve. Where the variability is very high, for instance in the first few milliseconds post-stimulus in the case of Poisson-like noise, the best-encoded stimulus is determined by the characteristic stimulus (cutoff point) of the tuning curve, which paradoxically can mean that an absence of activity conveys the most information in terms of estimation; this is not the case for peaked tuning curves.

We next examined how the behavioral task can affect the best-encoded stimulus. One of the strengths of the SSI is its flexibility: it can be used to analyze an arbitrary task by manipulating the stimulus distribution. Butts and Goldman (Butts and Goldman, 2006) showed that when the SSI is used to quantify information in the context of a specific task, the task itself can determine what the best-encoded stimulus is, with population size and variability having little effect. Our results support this and show that it also holds for monotonic tuning curves. In general, if tasks are thought of as lying on a continuum between the two extremes of stimulus distribution richness (i.e., two discrete stimuli and a continuous stimulus distribution), the simpler the task, the more influence it will have on the best-encoded stimulus. Where the best encoded stimulus is determined mainly by the task, population size and variability have little effect and no distinct asymptotic and non-asymptotic regimes exist (such as the peak and flank coding regimes for unimodal tuning curves). For fine discrimination tasks, the best-encoded stimuli are as predicted by the Fisher information: at the steepest parts of the tuning curve. As a rule of thumb, fine discrimination tasks are those where the stimulus spacing is less than the width of the tuning curve flank or flanks. For greater stimulus spacings the best-encoded stimuli shift from the flanks to the peak, or the ends of the flank for monotonic tuning curves. For tasks involving more than two distinct stimuli, the distance between the closest pair of stimuli in an ensemble is important, as it determines whether the fine discrimination regime is relevant. In addition to this, the closest stimuli are likely to have the most similar response distributions, which in turn determines the rate at which the mutual information increases with population size (Kang and Sompolinsky, 2001).

Although the SSI can be used to analyze many different tasks, using it to determine best-encoded stimuli in a two-alternative task is not computationally efficient. This type of analysis boils down to measuring the difference between two response distributions, and if these distributions are known—as in a modeling study—then a measure such as the Chernoff distance is more efficient. Our results confirm that the SSI and Chernoff distance are in close agreement as to the best-encoded stimuli in two-alternative tasks where each stimuli is equally likely. This is related to the work of Kang et al. (2004), who used Chernoff distance to analyze the relative amounts of information conveyed by neurons in discrimination tasks of varying coarseness. Whereas they constructed curves of Chernoff distance vs. stimulus interval for whole populations, we used it to identify which stimuli were best encoded by a given neuron. Just as the SSI and the Chernoff distance are consistent with one another where both are valid (two-alternative tasks), the SSI and the Fisher information are consistent where the Fisher information is valid, i.e., in the asymptotic regime and for fine discrimination tasks. The Fisher information gives insight into fine discrimination, the Chernoff distance into two-alternative discrimination of arbitrary coarseness, while the SSI is flexible and can give a generalized picture of the informational tuning curve. These measures do not contradict each other, they simply have limited validity and may not yield meaningful predictions outside their respective domains of validity.

Like any study, ours has a few limitations. Firstly, our model only considers information transmitted in firing rates, and assumes uncorrelated Poisson trial-to-trial variability. These are clearly simplifications that discount any information conveyed by spike times, but analysis of rate coding remains important as tuning curves are still widely used by experimentalists to describe the information-bearing activity of neurons. Also, correlations in trial-to-trial variability have been shown to have a relatively small effect on best-encoded stimuli (Yarrow et al., 2012). We have assumed that all tuning curves in a population are stereotypical copies with equal amplitude. Heterogeneity in the amplitude of tuned responses can have a significant effect on the total information content of a population code (Ecker et al., 2011), so it may also affect best-encoded stimuli; this should be investigated in future work.

In this article we have examined two classes of one-dimensional tuning function: unimodal and monotonic. In reality these are often encountered as a combined tuning function of two or more stimulus dimensions, for example a neuron in the visual cortex may be unimodally tuned to contour orientation and also monotonically responsive to contrast. Investigating the best-encoded stimuli in population codes comprised of neurons with two-dimensional combined monotonic and unimodal tuning functions would be an interesting topic for future research.

Estimating the best-encoded stimulus of a neuron is not an empty theoretical exercise; there is a growing body of evidence that the neurons with the most informative activity contribute strongly to decision-making. For any given task, determining the best-encoded stimulus of a given neuron, and identifying the neurons in a population that are most informative, are closely related problems (in fact they are exactly equivalent when all tuning curves are identical, shifted copies and the stimulus distribution is uniform). Testing the hypothesis that the most informative neurons contribute most to decision-making is relatively tractable for two-alternative tasks where the best-encoded stimulus is controlled by the task itself and behavioral performance in the task is easily measured. In a theoretical study of maximum-likelihood decoding of population codes, Jazayeri and Movshon (2006) found that the spacing between stimuli in a discrimination task was an important determinant of which neurons contribute to decision-making, with neurons tuned to the task stimuli contributing most to coarse discrimination and flanking neurons (i.e., those whose tuning curve flanks span the stimuli to be discriminated) contributing most to fine discrimination. The question of whether subsequent neural processing makes optimal use of the information propagated by a population code is an important one, as the information content of the population activity itself is less relevant if it is not fully utilized. Some experimental studies have reported evidence of population codes that are utilized optimally, in that the most informative neurons appear to have the greatest causal effect on behavior in both fine and coarse discrimination tasks. Evidence that flanking, “off-channel” neurons are most important in fine discrimination tasks has been found in psychophysical studies (e.g., Hol and Treue, 2001) and studies involving direct measurement of single neuron activity (Purushothaman and Bradley, 2005) and BOLD response in fMRI voxels (Scolari and Serences, 2010). Recent theoretical progress in the interpretation of choice probabilities (Haefner et al., 2013) opens the door to more robust estimation of neuronal contributions to decision making. Given these advances, we might expect to see new experimental evidence of the importance of flanking neurons in fine discrimination tasks, and unimodally-tuned neurons selective for the stimuli in coarse discrimination tasks—if firing rate information is indeed optimally utilized. Similarly, the results of our simulations suggest that neurons whose activity is just above baseline, or just reaching its saturated plateau level, at the stimulus of interest will be influential in coarse discrimination tasks involving monotonically-tuned populations.

Comparing precision at the neuronal and behavioral levels is much more difficult when the stimulus ensemble is richer than that of a simple two-alternative discrimination task, for instance in the case of estimation. This makes it difficult to test whether the pre-asymptotic regime (where the best encoded stimuli do not coincide with the flanks of the tuning curves) is biologically relevant. Our results confirm that the pre-asymptotic regime is restricted to very high levels of trial-to-trial variability or very short integration times when the population size is of the order of hundreds of neurons, as it likely to be the case in the cortex. Do neural systems ever operate in the pre-asymptotic regime? If they do, is subsequent information processing adapted to make use of the fact that different neurons may be most informative at short vs. long integration times? These remain open questions. It may be important to consider stimulus detection when addressing these questions, as the time required to accumulate evidence for detection may impose a lower limit on the range of integration times that are relevant for estimation.

To conclude, our results should serve as a reminder that it is not safe to assume that strong neuronal responses are informative; it is perhaps more often the case that moderate responses are most informative, as these occur in response to stimuli that lie on the flanks of the tuning curve. However, information tuning curves and best-encoded stimuli can be easily estimated from experimentally measured tuning curves using the measures discussed in this article.

## Author contributions

This study was conceived by SY and PS. SY designed and carried out the simulations, analyzed the results and prepared the figures. The article was written by SY and PS.

### Conflict of interest statement

The authors declare that the research was conducted in the absence of any commercial or financial relationships that could be construed as a potential conflict of interest.
